# Dual-modified cationic liposomes loaded with paclitaxel and survivin siRNA for targeted imaging and therapy of cancer stem cells in brain glioma

**DOI:** 10.1080/10717544.2018.1494225

**Published:** 2018-10-01

**Authors:** Xiyang Sun, Ying Chen, Hui Zhao, Guanglei Qiao, Meiyang Liu, Chunlei Zhang, Daxiang Cui, Lijun Ma

**Affiliations:** a Department of Oncology, Tongren Hospital, Shanghai Jiao Tong University School of Medicine, Shanghai, PR China;; b Department of Geriatrics, Tongren Hospital, Shanghai Jiao Tong University School of Medicine, Shanghai, PR China;; c Institute of Nano Biomedicine and Engineering, Shanghai Engineering Research Center for Intelligent Instrument for Diagnosis and Therapy, Department of Instrument Science and Engineering, Shanghai Jiao Tong University, Shanghai, PR China;; d National Center for Translational Medicine, Collaborative Innovational Center for System Biology, Shanghai Jiao Tong University, Shanghai, PR China

**Keywords:** Glioma stem cells, survivin siRNA, paclitaxel, targeted imaging, targeted therapy

## Abstract

Development of safe, efficient nanocomplex for targeted imaging and therapy of cancer stem cells in brain glioma has become a great challenge. Herein, a low-density lipoprotein receptor-related protein and a RNA aptamer bound CD133 were used as dual-targeting ligands to prepare dual-modified cationic liposomes (DP-CLPs) loaded with survivin siRNA and paclitaxel (DP-CLPs–PTX–siRNA) for actively targeting imaging and treating CD133^+^ glioma stem cells after passing through the blood–brain barrier. After being administrated with DP-CLPs–PTX–siRNA nanocomplex, DP-CLPs showed a persistent target ability to bind glioma cells and brain microvascular endothelial cells (BCECs) and to deliver drugs (PTX/siRNA) to CD133^+^ glioma stem cells. Prepared DP-CLPs–PTX–siRNA nanocomplex showed very low cytotoxicity to BCECs, but induced selectively apoptosis of CD133^+^ glioma stem cells, and improved CD133^+^ glioma stem cells' differentiation into non-stem-cell lineages, also markedly inhibited tumorigenesis, induced CD133^+^ glioma cell apoptosis in intracranial glioma tumor-bearing nude mice and improved survival rates. In conclusion, prepared DP-CLPs–PTX–siRNA nanocomplex selectively induced CD133^+^ glioma stem cell apoptosis *in vitro* and *in vivo* exhibits great potential for targeted imaging and therapy of brain glioma stem cells.

## Introduction

Glioblastoma multiform (GBM) is the most common and aggressive primary tumor of the central nervous system, presenting extremely high mortality rates. In spite of surgical resection in conjunction with current adjuvant therapies, the median survival period for GBM patients is presently only 14.6 months. This is mainly due to interference caused by the existence of blood–tumor barrier (BTB) and blood–brain barrier (BBB), which hampers tumor accumulation and uptake of therapeutic agents (Guo et al., 2011; Yang et al., 2014). Cancer stem cells (CSCs), a cancer cell subpopulation with high self-renewal and stemness properties, are responsible for cancer metastasis, relapse and chemoradioresistance (Cheng et al., 2010; Alama et al., 2012). Conventional therapeutic agents are able to target and eliminate the tumor mass but lack effectiveness against CSCs, contributing to cancer relapse (Chiou et al., 2012). Therefore, it is fundamentally important to develop novel therapeutic strategies that selectively target CSCs to improve cancer treatment outcome (Zhao et al., 2013; Wang et al., 2015; Shen et al., 2016). Recently, various types of nanoparticles have been designed with the aim of effectively inhibiting CSCs. These nanoparticles target specific signaling pathways and/or biomarkers, which are critically involved in malignant tumor development and maintenance (Leon et al., 2016; Zhao et al., 2016). However, due to the anatomical location of gliomas and their propensity for aggressive growth, specifically delivering agents to glioma stem cells (GSCs) has proven challenging. Additionally, distinguishing between CSCs and nonmalignant normal stem cells represents another large obstacle (Yu et al., 2012; Kreso et al., 2014).

CD133, a recognized key CSC marker, is particularly for glioblastoma. CD133^+^ cells derived from glioblastomas display CSC-like properties and are resistant to currently used conventional therapies, including radiation and chemotherapy with a high rate of recurrence (Shigdar et al., 2013). A15, a RNA aptamer that binds to CD133, can be internalized efficiently after binding, which has been successfully exploited as a targeting ligand for tracking CD133^+^ cancer cells (Jianxin Jiang et al., 2015). Additionally, it has been previously demonstrated that angiopep-2 can actively penetrate into the brain compartment by targeting low-density lipoprotein receptor-related protein (LRP), which is highly expressed on the surface of the BBB, angiopep-2 was able to enhance drug delivery across the BBB and BTB to glioma cells (Ren et al., 2012). Accordingly, angiopep-2 and A15 can be potentially adopted as dual-targeting ligands capable of crossing BBB/BTB and tracking CD133^+^ cancer cells in gliomas.

Accumulating evidence has suggested that GSCs contribute to the therapy resistance by preferentially upregulating the DNA damage checkpoint proteins. Once chemoradiotherapy cause DNA damage, the checkpoint proteins are activated more efficiently in GSCs compared with non-GSCs, which presently suppress apoptosis by facilitating the DNA repair process following therapeutic injuries. (Bao et al., 2006). Survivin is an inhibitor of apoptosis (IAP) that is an important group of proteins involved in regulation of apoptosis. Survivin is strongly expressed in GBM tissues, particularly in patient-derived CSC cultures, and its expression is significantly higher in recurrent GBM versus the newly diagnosed GBM (Hacer Guvenc et al., 2013; Serena Acquati et al., 2013). Thus, survivin may be an ideal molecular target for novel cancer therapeutic strategies, especially in CSCs-targeting therapy (Dahan et al., 2014; Hadi AlShamaileh et al., 2017). Synergistic paclitaxel (PTX) plus survivin siRNA combination represents a potentially useful chemo-gene therapeutic strategy (Shen et al., 2012; Jianan Shen et al., 2014; Chen et al., 2017). Previous research has demonstrated that PTX administration alone significantly induced survivin protein expression in residual tumor cells. Inhibiting survivin upregulation completely reversed PTX induction of survivin expression and enhanced PTX activity (Kar et al., 2015; Salzano et al., 2015). However, thus far, the effectiveness of combination therapy of PTX and survivin siRNA on GSCs has not been reported.

In the current study, the dual-modified cationic liposomes (DP-CLPs) were designed by attaching two receptor-specific ligands, angiopep-2 and A15. DP-CLPs were used to deliver a combination of survivin siRNA and PTX (DP-CLPs–PTX–siRNA) directly and selectively to glioma cells and especially glioma stem cells. This study investigated the *in vitro* and *in vivo* targeting efficiency and the pharmacodynamics of DP-CLPs–PTX–siRNA nanocomplex, as well as its effect on CSC survival and brain glioma growth.

## Materials and methods

### Materials

Angiopep-2 (TFFYGGSRGKRNNFKTEEY) was synthesized by Shanghai Gene-Pharma Co. Ltd. (Shanghai, China). A15 aptamers (sequence: 5′-NH2-CCCUCCUACAUAGGG-3′) were synthesized by Shanghai Gene-Pharma Co. Ltd. DC-chol, DOPE, rhodamine-DOPE and COOH-PEG_2000_-DSPE were provided by Avanti Polar Lipids (Alabaster, AL, USA). Survivin siRNA (sequence: 5′-GCAUUCGUCCGGUUGCGCUdTdT-3′) and a scrambled siRNA (sequence: 5′-AUGAACUUCAGGGUCAGCUdTdT-3′) were purchased from Thermo Scientific Dharmacon (Shanghai, China). The following primer probe sets (Integrated DNA Technologies, Coralville, IA, USA) were used: survivin, forward: 5′-CAACCGGACGAATGCTTTT-3′; reverse: 5′-AAGAACTGGCCCTTCTTGGA-3′; probe: 5′-/5HEX/CCAGATGAC/ZEN/GACCCCATAGAGGAA/3IABkFQ/-3′; GAPDH, forward: 5′-AATCCCATCACCATCTTCCAG-3′; reverse: 5′-AAATGAGCCCCAGCCTTC-3′; probe: 5′-/5Cy5/CCAGCATCGCCCCACTTG ATTTT/3IAbRQSp/-3′; β-actin primers, forward: 5′-CATCGTGGGCCGCCCTAGGC-3′, reverse: 5′-GGGCCTCGGTGAGCAGCACA-3′ (Sangon Biotech, Shanghai, China). Paclitaxel was purchased from Fujian South Bio-Engineering Co. Ltd. (Fujian, China). Survivin, nestin, GFAP, BCRP1 and MGMT antibody were obtained from Cell Signaling Technology (Danvers, MA, USA). E.Z.N.A.^®^ HP Total RNA Kits were purchased from Omega Biotek (Norcross, GA, USA), and qScript™ cDNA SuperMix and PerfeCTa^®^ MultiPlex qPCR SuperMix were obtained from Quanta Biosciences (Gaithersburg, MD, USA). Temozolomide capsules were purchased from Jiangsu Tasly Diyi Pharmaceutical Co. Ltd (Jiangsu, China). 1,1′-Dioctadecyl-3,3,3′,3′-tetramethyl indotricarbocyanine iodide (DiR) was offered by Biotium (Hayward, CA, USA). Cell counting kit-8 (CCK8) was obtained from Dojindo Laboratories (Kumamoto, Japan), and Annexin V-FITC Apoptosis Detection Kits were obtained from BD Pharmingen (Heidelberg, Germany). CD133 MicroBead Kit, as well as anti-human CD133 and phycoerythrin (PE)-labeled CD133/2 (293C3) antibodies (PE-CD133 antibodies), was obtained from Miltenyi Biotec (Bergisch Gladbach, Germany). IRDyeTM800 conjugated anti-goat and anti-rabbit second antibodies were obtained from Rockland Inc. (Limerick, PA, USA). DMEM-F12 and other cell culture media were provided by Gibco-BRL (Gaithersburg, MD, USA). Human recombinant bFGF, EGF and N2 supplements were obtained from R&D (Minneapolis, MN, USA). All the other chemicals used were of analytical or high-performance liquid chromatography (HPLC) grade.

### Animals

Male BALB/c nude mice (18–20 g) were purchased from the Shanghai Experimental Animal Center (Shanghai, China). Animal experiments were carried out in accordance with protocols evaluated and approved by the Ethical Committee of Shanghai Jiao Tong University.

### Cell lines

U251 cells were obtained from the Institute of Biochemistry and Cell Biology, Shanghai Institutes for Biological Sciences, Chinese Academy of Sciences (Shanghai, China). Brain capillary endothelial cells (BCECs) were purchased from Cell Bank, Chinese Academy of Sciences (Shanghai, China). Both cell types were cultured in DMEM supplemented with 10% FBS, 1% nonessential amino acids, 100 IU/ml of penicillin and 100 mg/ml of streptomycin sulfate. CD133^+^ glioma cells were cultured in stem cell growth medium (STGM; composed of DMEM/F12, B27 supplement, penicillin and streptomycin, 20 ng/ml recombinant basic fibroblast growth factor (bFGF), 20 ng/ml epidermal growth factor (EGF)) at relatively low densities (1–3 × 10^5^ cells/ml) in T25 tissue culture flasks. All cells were cultured in incubators maintained at 37 °C with 5% atmospheric CO_2_ under fully humidified conditions.

### CSC isolation and characterization

CD133^+^ glioma cells were isolated using the Miltenyi Biotec CD133 isolation kit. First, U251 cells cultured in stem cell growth medium were enriched for CD133^+^ cells by using ultra-low adhesion flasks. Floating tumor spheres were extracted, disaggregated into single cells and characterized via staining with CD133/2-APC or isotype control antibody and subsequent flow cytometric analysis using a BD FACSCalibur (BD Biosciences, San Jose, CA, USA). Sterile aliquots of CD133^+^ cells were resuspended in STGM and maintained. To isolate adherent CSCs, culture flasks were coated with 100 μg/ml poly-d-lysine (Sigma) for 1 h and then coated with 10 μg/ml laminin (Invitrogen) for 2 h prior to use. Adherent CSCs were dissociated with HyQTase (Thermo Scientific) and split 1:3. Under these conditions, the CSCs grew in an adherent monolayer, maintaining their CD133 expression and stem-like characteristics.

WST-1 cell proliferation assay was utilized to examine drug sensitivity. Both U251-CD133^+^ and U251-CD133^–^ subsets were exposed to PTX at various concentrations for up to 24 h in respective culture media.

BCRP1 has been shown to play an important role in the drug resistance of normal stem cells and CSCs (Xin et al., 2012). The DNA repair protein MGMT has been proved to render cells resistant to the cytotoxic actions of methylating and chloroethylating agents such as PTX (Vlaming et al., 2015). Survivin, nestin, GFAP, BCRP1 and MGMT protein expression levels in U251-CD133^+^ and U251-CD133^–^ subsets were detected by western blotting.

### *Preparation and characterization of DP-CLPs*–*PTX*–*siRNA nanocomplex*

DSPE-PEG_2000_-angiopep and DSPE-PEG_2000_-A15 were synthesized by coupling the targeted ligand (angiopep-2 and A15, respectively) with the COOH group of COOH-PEG_2000_-DSPE using EDC as a coupling agent. Adequate amounts of EDC and NHS (COOH-PEG_2000_-DSPE:EDC:NHS ratio =0.03:1.25:2.1 μmol/μmol) were added as previously described (Sun et al., 2012). Unmodified CLPs (cationic liposomes) and DP-CLPs were prepared as previously described with minor modifications. Briefly, the lipid components DC-chol, DOPE and DSPE-PEG_2000_-angiopep or DSPE-PEG_2000_-A15 were dissolved with chloroform (or containing PTX, DiR) in a glass flask at a molar ratio of 2:4:0.03:0.03. 0.03 μmol of angiopep-2 or A15 was added, the final lipid concentration of DP-CLPs–PTX–siRNA was 20 mg/ml. The chloroform was then evaporated to dryness under vacuum using a rotary evaporator (Buchi AG, Flawil, Switzerland) for 2 h at 40 °C. The dried lipid film was subsequently hydrated using RNase-free buffer for 30 min. After ultrasonication for 10 min using a probe-type ultrasonicator, the lipid dispersion was sequentially extruded polycarbonate filters using an Avanti Polar Lipids Mini-Extruder. Unencapsulated DiR or PTX was removed by passing the liposome suspension through a Sepharose CL-4B gel column. DP-CLPs–PTX–survivin siRNA or CLPs–PTX–survivin siRNA were formed by gently mixing DP-CLPs/PTX or CLPs/PTX with an aqueous survivin siRNA solution (10 μM) and incubating for 30 min at 20 °C. The negative control DP-CLPs–scrambled siRNA or CLPs–scrambled siRNA was formed by mixing DP-CLPs or CLPs with an aqueous scrambled siRNA solution (10 μM).

Agarose gel electrophoresis was carried out to determine the siRNA (survivin siRNA or scrambled siRNA) loading capabilities of DP-CLPs/PTX or CLPs/PTX at lipid/siRNA ratios 1:0.05, 1:0.08, 1:0.1, 1:0.15 and 1:0.2 (w/w). The samples were then analyzed by electrophoresis using 2% agarose gels to monitor siRNA release. The gel was run at 120 V for 15 min and subsequently imaged using an ODYSSEY infrared imaging system.

Particle size distribution and zeta potential were measured using a dynamic light scattering detector (Zetasizer, Nano-ZS; Malvern Instruments, Malvern, UK). Size changes before and after BSA addition (10%) were also measured simultaneously. A nanoscope atomic force microscope (Digital Instruments, Santa Barbara, CA, USA) was utilized to collect images.

The drug loading coefficients (DL%) and encapsulation ratios (ER%) of DP-CLPs–PTX–siRNA were investigated. PTX or DiR *in vitro* release behavior was monitored using a previously described dialysis method (Kitange et al., 2009). PTX concentrations in samples were determined using HPLC as described earlier with corrections for volume replacement. DiR concentrations in samples were determined using a fluorospectrophotometer (F-1000; Hitachi, Tokyo, Japan).

### Cellular uptake

Rhodamine-DOPE was introduced for lipid composition labeling to evaluate cellular uptake. U251-CD133^+^ cells, U251-CD133^–^ cells and BCECs were seeded at a density of 5 × 10^4^ cells/well in six-well plates. After 48 h incubation in their own respective culture conditions, cells were examined for confluency and morphology using the light microscopy. They were then incubated with either DP-CLPs–PTX–survivin siRNA, CLPs–PTX–survivin siRNA and the control (DP-CLPs–PTX–scrambled siRNA and the CLPs–PTX–scrambled siRNA) at quantities required to deliver 10 μg of lipids per well in the presence of serum-free medium for 60 min at 37 °C. Cells were then washed with Hanks’ balanced salt solution and visualized and photographed using an IX2-RFACA fluorescent microscope (Olympus, Osaka, Japan).

### qPCR analysis

qPCR was used to determine cellular survivin gene expression after treatment with DP-CLPs–PTX–siRNA. U251-CD133^+^ cells, U251-CD133^–^ cells and BCECs were seeded (5 × 10^4^ cells/well) in six-well plates and treated with the following formulations at 37 °C for 48 h: DMEM, DP-CLPs–scrambled siRNA, CLPs–scrambled siRNA (control), DP-CLPs–PTX–survivin siRNA and CLPs–PTX–survivin siRNA (in the quantity required to deliver 100 nM siRNA, 200 ng PTX). Total RNA was extracted using the E.Z.N.A.^®^ HP Total RNA Kit and reverse transcribed to cDNA using qScript™ cDNA SuperMix. Real-time RT-PCR (triplicate samples, 5 μl cDNA per reaction) was performed with PerfeCTa^®^ MultiPlex qPCR SuperMix (Quanta Biosciences) in a CFX96™ Real-Time PCR Detection System (Bio-Rad, Hercules, CA, USA). The multiplex thermal reaction program used was as follows: 3 min at 95 °C followed by 40 cycles of 15 s at 95 °C and 1 min at 60 °C. mRNA expression relative to GAPDH expression was calculated using the ΔΔCt method.

### Western blot analysis

To detect survivin protein expression in U251-CD133^+^ cells, U251-CD133^–^ cells and BCECs treated with either DP-CLPs–scrambled siRNA (negative control) or DP-CLPs–PTX–survivin siRNA (in the quantity required to deliver 100 nM siSurvivin, 200 ng PTX), western blotting was performed. Cells were seeded (5 × 10^4^ cells/well) in six-well plates and incubated for 48 h. The cells were then washed twice with PBS and lysed in lysis buffer for 30 min. Whole cell lysates were centrifuged at 12,000 rpm for 5 min. The supernatant was then extracted and transferred to a new tube for measuring protein concentrations using a BCA (bicinchoninic acid) protein assay kit. To perform immunoblotting, protein samples were loaded onto a 15% SDS-polyacrylamide gel, separated via electrophoresis (SDS-PAGE) and transferred onto PVDF membranes (Millipore, Bedford, MA, USA). Immune complexes were formed by incubating membranes with primary antibodies overnight at 4 °C. Blots were then washed and incubated for 1 h with IRDye™800 conjugated anti-goat and anti-mouse secondary antibodies. Immunoreactive protein bands were detected using an Odyssey Scanning System (LI-COR, Lincoln, NE, USA).

### Antiproliferation assay

The antiproliferation activity was evaluated using a CCK8 assay. Briefly, U251-CD133^+^ cells, U251-CD133^–^ cells and BCECs cells were seeded in 96-well plates at a density of 5,000 cells/well or 50 spheres per well. After 12 h, cells were treated with DMEM, DP-CLPs–scrambled siRNA or CLPs–scrambled siRNA as controls, or DP-CLPs–PTX–survivin siRNA, or CLPs–PTX–survivin siRNA in sufficient quantities to deliver 100 nM survivin siRNA and 200 ng PTX. Forty-eight hours later, 10 ml of CCK8 was added to each well and incubated for 30 min. The plates were then subjected to a microplate reader (Thermo Multiskan MK3, Shanghai, China) for cell viability assay at the wavelength of 450 nm.

### *In vitro* quantitative apoptosis detection

U251-CD133^+^ cells, U251-CD133^–^ cells and BCECs were seeded at a density of 5 × 10^4^ cells/well in a sic-well plate. Culture media were then substituted with DMEM, DP-CLPs–scrambled siRNA or CLPs–scrambled siRNA as controls, or DP-CLPs–PTX–survivin siRNA siRNA or CLPs–PTX–survivin siRNA siRNA in sufficient quantities to deliver 100 nM of survivin siRNA and 200 ng of PTX. After 48 h incubation, the cells were trypsinized, centrifuged at 1,000×*g* for 5 min and treated using an Annexin V-FITC apoptosis detection kit. Apoptotic cells were quantified using a FACS scan Flow Cytometer (BD Pharmingen).

### *In vivo* bioluminescence imaging

U251-CD133^+^ cells (5.0 × 10^5^ cells suspended in 5 μl PBS) were implanted in male Balb/c nude mice at a location 1.8 mm lateral to the bregma and at 3 mm of depth using a stereotactic fixation device with a mouse adapter. At 16 days postimplantation, intracranial U251-CD133^+^ glioma-bearing mice were injected via tail vein with 100 μl DiR-labeled DP-CLPs–PTX–survivin siRNA or CLPs–PTX–survivin siRNA with a final dosage of 2.5 μM survivin siRNA/mouse, as well as 5 μg PTX/mouse. At 24 h post iv, mice were anesthetized with ip administered 10% chloral hydrate and placed on an animal plate heated to 37 °C. Fluorescence emissions from 620 to 900 nm were captured using an *in vivo* imaging system (CRi, Woburn, MA, USA).

To compare the tissue and tumor distributions of DP-CLPs–PTX–survivin siRNA and CLPs–PTX–survivin siRNA, the tumor-bearing brain as well as major organs, including the liver, lungs, spleen, kidneys and heart, were dissected, washed with saline and subjected to *ex vivo* fluorescence imaging.

### *In vivo* tumor formation ability

U251-CD133^+^ cells were seeded at a density of 5 × 10^4^ cells/well in a six-well plate, STMG culture media was substituted with DP-CLPs–scrambled siRNA, CLPs–scrambled siRNA (controls), DP-CLPs–PTX–survivin siRNA or CLPs–PTX–survivin siRNA (in appropriate quantities required deliver 100 nM survivin siRNA and 200 ng PTX). After 48 h incubation, cells were extracted and suspended in 5 μl of PBS and then implanted into the right striatum of male Balb/c nude mice at a point 1.8 mm lateral to the bregma and to a depth of 3 mm using a stereotactic fixation device with a mouse adapter. Mice were scanned using 1.5 T MRI at 19 days postimplantation and then sacrificed in order to measure tumor size (*n* = 5/group).

### *In situ* tumor apoptosis detection and survival monitoring

At 7, 9, 11 and 13 days postimplantation of U251-CD133^+^ cells, mice were dosed with either DP-CLPs–PTX–survivin siRNA or CLPs–PTX–survivin siRNA via tail vein injection in quantities required to deliver 2.5 μM of survivin siRNA and 5 μg of PTX. Mice were sacrificed on day 16 postinjection, with the remaining mice monitored for survival duration. Frozen tissue sections (20 μm thick) were prepared from the sacrificed mice. Apoptotic cell death in tumor tissues was detected using the Annexin V-FITC Apoptosis Detection kit in accordance with manufacturer’s instructions and then visualized under the fluorescence microscopy.

### HE staining of important organs

On days 0, 7 and 21, the nude mice treated with DP-CLPs were sacrificed and the different organs were separated. All excised organs were washed twice with PBS and stored in 4% formalin. Then, they were further processed and stained with hematoxylin and eosin (H&E) for histological analysis.

### Statistical analyses

All experiments were performed at least three times with representative results presented (quantitative data is expressed as the mean ± SD). Statistical comparisons were made using Student's *t-*test. Survival analysis was computed by the Kaplan–Meier method and compared using the log-rank test.

## Results and discussion

### Isolation and characterization of CSCs

In the present study, U251 cells cultured in STGM and formed cell spheres after 3 days. After forming spheres of at least 100 cells (culturing for approximately 5 days) and 15 days later, there were all floating tumor spheres (Supplementary Figure S1(A)), which highly expressed CD133 (Supplementary Figure S1(B)). Compared with the cells cultured in DMEM medium, which contained 6.7% CD133^+^ cells (Supplementary Figure S1(C-a)), the cells cultured in STGM for 15 days contained a higher percentage of CD133^+^ cells 24.9% (Supplementary Figure S1(C-b)). U251 cells were cultured in STGM for enrichment before magnetic separation of CD133^+^ cells. We found that U251-CD133^+^ showed dramatic drug resistance to PTX at different concentrations compared to U251-CD133^–^ (Supplementary Figure S1(D)), and U251-CD133^+^ had much higher expression of survivin protein, neural stem cell marker Nestin and drug resistance-related protein BCRP1, MGMT, but lacked of neural differentiation marker GFAP compared with U251-CD133^–^ (Supplementary Figure S1(E)). It suggested that the isolated U251-CD133^+^ exhibit the characteristics of stem cells and can be adopted as a CSC model for the subsequent experiments.

### Characterization

**Figure 1.**
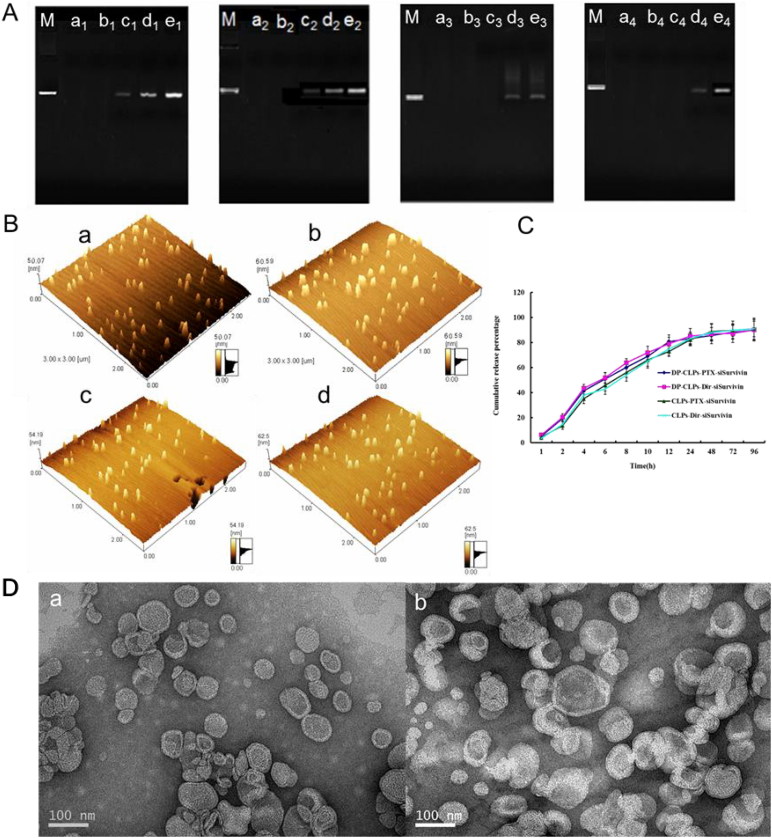
Characterization of nanocomplex. (A) Agarose gel electrophoretic mobility shift assay was performed for DP-CLPs–PTX–survivin siRNA (M-e^1^), DP-CLPs–scrambled siRNA (M-e^2^), CLPs–PTX–survivin siRNA (M-e^3^) and CLPs–scrambled siRNA (M-e^4^). Control was in lane M. Lanes a, b, c, d and e corresponded to lipid/siRNA ratios of 1:0.05, 1:0.08, 1:0.1, 1:0.15 and 1:0.2 (w/w), respectively. (B) Atomic force microscopy pictures of (a) CLPs–scrambled siRNA, (b) DP-CLPs–scrambled siRNA, (c) CLPs–PTX–survivin siRNA siRNA and (d) DP-CLPs–PTX–survivin siRNA siRNA at a lipid/siRNA weight ratio of 1:0.08. (C) PTX and DiR *in vitro* release profiles from the above liposomes. (D) TEM of (a) CLPs–PTX–survivin siRNA siRNA and (b) DP-CLPs–PTX–survivin siRNA siRNA at a lipid/siRNA weight ratio of 1:0.08.

CLPs and DP-CLPs produced sufficient siRNA retardation at a lipid/siRNA weight ratio of 1:0.08 (Figure 1(A)). Thus, this ratio was used in subsequent experiments in order to maximize drug loading. Quantitative analyses of particle size and zeta potential are shown in Table 1. Upon conjugation with angiopep-2 or A15, CLPs average diameter increased slightly while zeta potential showed a decrease in surface charge. Upon addition of siRNA, both DP-CLPs and CLPs showed increased average diameters and decreased surface charge. It suggested that conjugation with angiopep-2 and A15 induces the decrease in positive potential and results in a lower likelihood for CLPs to integrate with BSA, and addition of siRNA can also minimize interactions with serum proteins. The polydispersity of all the formulations also showed a narrow size distribution (PDI <0.28), and DP-CLPs–PTX–siRNA nanocomplex with lipid/siRNA ratios of 1:0.08 were smaller than 135 nm in mean diameter following the addition of BSA and appeared spherical in shape with relatively monodispersed sizes (Figure 1(B,D)). All the factors showed that DP-CLPs–PTX–siRNA constructed in this work with a good biodistribution and plasma stability, may be more stable after injection.

**Table 1.** Size and zeta potentials of various particles (*n* = 4 or 5).

Formulation	Mean diameter in H_2_O (nm)	PDI	Mean diame ter in BSA (nm)	PDI	Zeta potential in H_2_O (mV)
CLPs	50 ± 4.6	0.117 ± 0.012	267.2 ± 35.7	0.259 ± 0.067	49.3 ± 7.6
DP-CLPs	59 ± 2.8	0.101 ± 0.010	248.2 ± 35.7	0.261 ± 0.078	44.8 ± 7.3
CLPs–scrambled siRNA	100.2 ± 5.9	0.133 ± 0.052	155.7 ± 10.2	0.149 ± 0.016	21.2 ± 0.7
CLPs–PTX–survivin siRNA	100.8 ± 7.2	0.121 ± 0.042	156.2 ± 24.1	0.179 ± 0.039	21.8 ± 5.6
DP-CLPs–scrambled siRNA	116.2 ± 5.1	0.193 ± 0.089	121.5 ± 10.9	0.139 ± 0.024	11.2 ± 0.9
DP-CLPs–PTX–survivin siRNA	118.7 ± 6.3	0.134 ± 0.082	119.5 ± 12.7	0.127 ± 0.032	11.5 ± 0.6

() represents the weight ratio of lipids and siRNA.

This profile confirmed that nanocomplex did not change DiR or PTX release behavior (Figure 1(C)). The PTX-loading coefficient of DP-CLPs–PTX–siRNA, at 1 mg added, was 2.5 ± 0.6% with a 97.1 ± 1.9% encapsulation efficiency. The PTX-loading coefficient of CLPs–PTX–siRNA, at 1 mg added, was 2.7 ± 0.4% with a 98.2 ± 0.6% encapsulation efficiency.

### Cellular uptake

**Figure 2.**
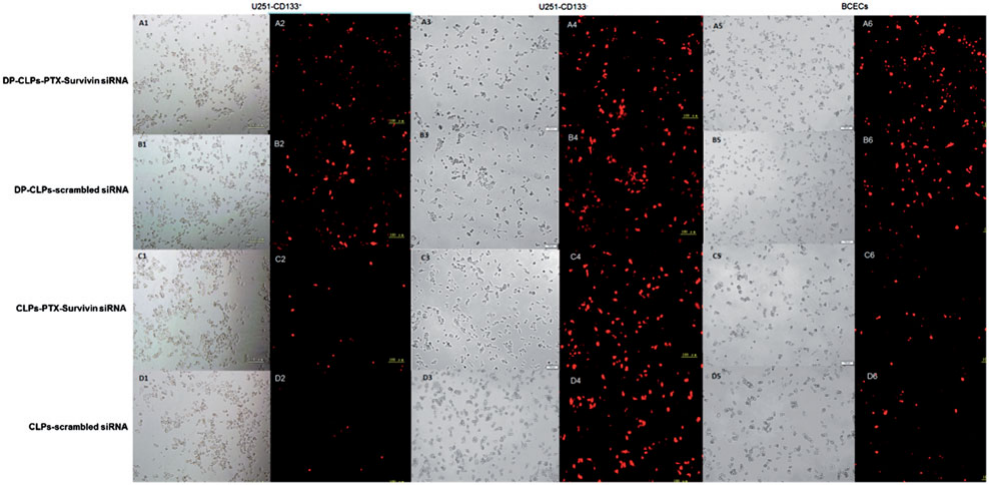
Cellular uptake. Cellular uptake of DP-CLPs–PTX–survivin siRNA (A1–A5), DP-CLPs–scrambled siRNA (B1–B5), CLPs–PTX–survivin siRNA (C1–C5) or CLPs–scrambled siRNA (D1–D5) particles (lipid/siRNA weight ratio of 1:0.08) by U251-CD133+ cells (A1–D1 and A2–D2), U251-CD133– cells (A3–D3 and A4–D4) or BCECs (A5–D5 and A6–D6) was examined by fluorescence microscopy after 60 min incubation. Phase contrast images were obtained before each corresponding fluorescent image. Red: rhodamine. Scale bar: 100 μm.

Cellular uptake of the modified group (DP-CLPs–PTX–survivin siRNA and DP-CLPs–scrambled siRNA) in U251-CD133^+^ cells and BCECs was enhanced compared to uptake of the unmodified group (CLPs–PTX–survivin siRNA or CLPs–scrambled siRNA) (Figure 2). DP-CLPs–PTX–siRNA persisted the binding ability to glioma cells and BCECs, this is mainly due to the dual targeting effect of angiopep-2 and DP-CLPs–PTX–siRNA especially facilitated drug into CD133^+^ glioma cells most due to the modification of A15.

**Scheme 1.**
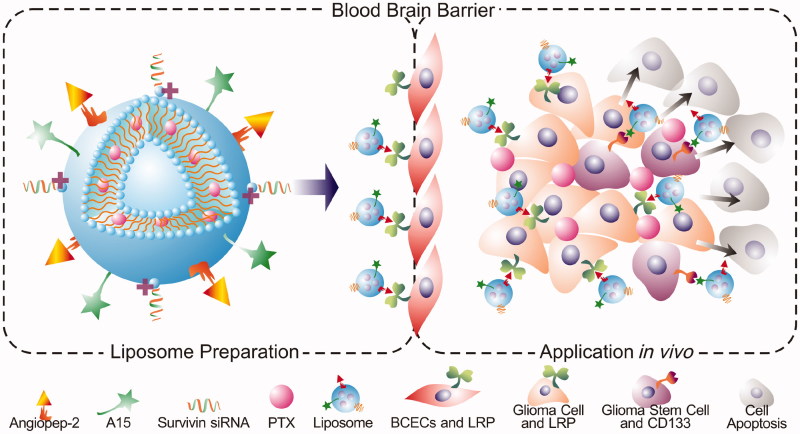
Schematic illustration of DP-CLPs–PTX–siRNA nanocomplex.

### *In vitro* analysis of survivin expression

Survivin mRNA levels were much higher in U251-CD133^+^ cells versus both U251-CD133^–^ cells and BCECs, while U251-CD133^–^ survivin mRNA levels were higher than that of BCECs (Supplementary Figure S2(A)). No significant expression downregulation was observed when U251-CD133^+^ cells, U251-CD133^–^ cells or BCECs were treated with survivin siRNA alone. However, survivin mRNA levels in U251-CD133^+^ cells decreased significantly when treated with DP-CLPs–PTX– survivin siRNA versus both DMEM-treated and CLPs–PTX– survivin siRNA-treated counterparts. In contrast, DP-CLPs–PTX–survivin siRNA and CLPs–PTX–survivin siRNA treatments resulted in similar decreases in survivin mRNA levels in U251-CD133^–^ cells. The silencing efficiency of siRNA against survivin was a comprehensive reflection of the delivery efficacy of DP-CLPs–PTX–siRNA. Generally, for U251-CD133^+^, U251-CD133^–^ with relative high expression mRNA level of survivin, the higher cellular uptake, as well as the preferable nuclear accumulation, the more robust inhibition of survivin was achieved, but for BCECs with inherent low expression mRNA level of survivin, there is poorer block effect, in spite of its higher uptake. Accordingly, Western blotting revealed that treatment with DP-CLPs–PTX–survivin siRNA decreased survivin protein expression in both U251-CD133^+^ and U251-CD133^–^ cells (Supplementary Figure S2(B)).

### *In vitro* antiglioblastoma activity

**Figure 3.**
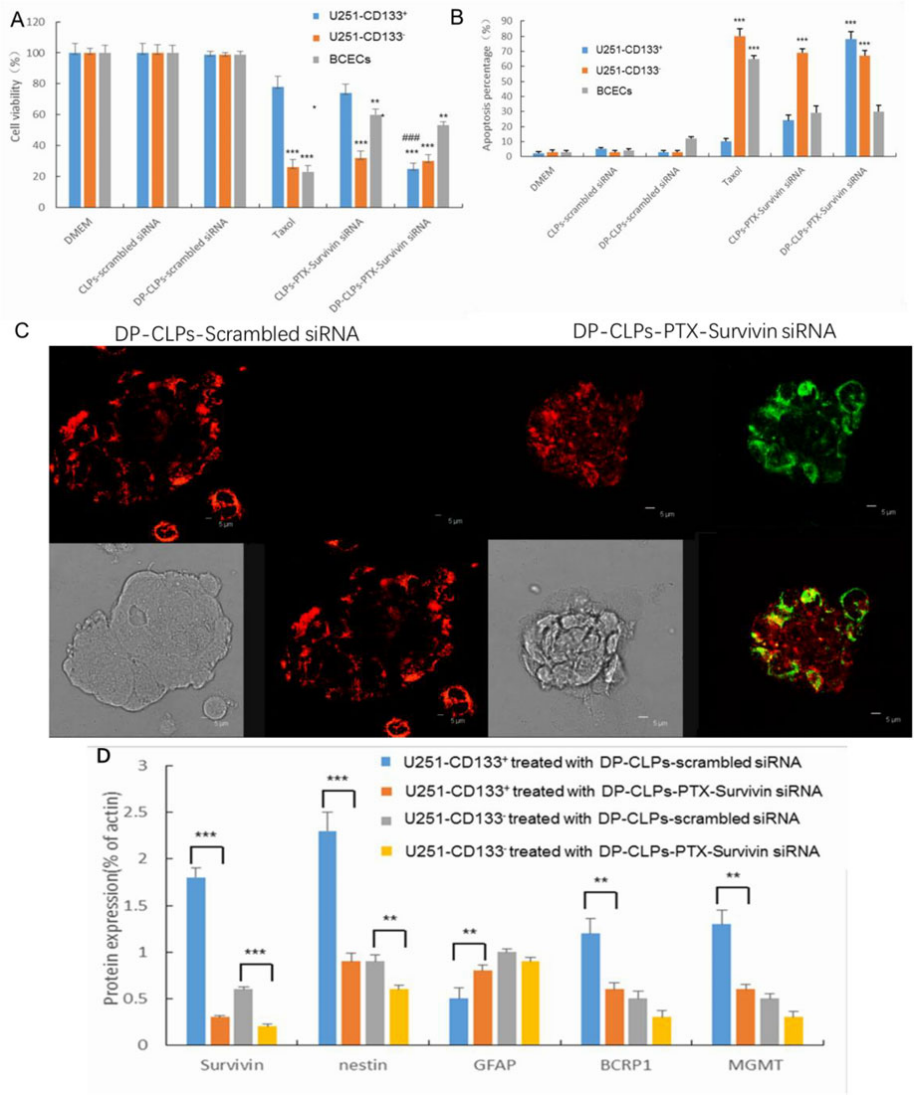
(A) Quantification of the viability of U251-CD133^+^ cells, U251-CD133^–^ cells and BCECs after treatment with DMEM, CLPs–scrambled siRNA, DP-CLPs–scrambled siRNA, PTX, CLPs–PTX–survivin siRNA siRNA or DP-CLPs–PTX–survivin siRNA for 48 h. *p < .05, **p < .01, ***p < .001 versus control; ^###^*p* < .001 versus the CLPs–PTX–survivin siRNA group. (B) Induction of apoptosis in U251-CD133^+^ cells, U251-CD133^–^ cells and BCECs following 48 h incubation with various siRNA formulations at siRNA concentrations of 100 nM. (C) Apoptosis of U251-CD133^+^ cells induced by 2 h treatment with either DP-CLPs–scrambled siRNA or DP-CLPs–PTX–survivin siRNA was examined using confocal microscopy after 48 h post-treatment incubation. Cells were treated using the Annexin V-FITC Apoptosis Detection Kit for 15 min to label apoptotic cells. Green: FITC-labeled phosphatidylserine in plasma membranes. Red: phycoerythrin. (D) Survivin, nestin, GFAP, BCRP1 and MGMT protein expression levels in U251-CD133^+^ and U251-CD133^–^ cells after 48 h treatment with DP-CLPs–scrambled siRNA or DP-CLPs–PTX–survivin siRNA. Data presented represent mean ± SD (*n* = 3). * *p* < .05, **p < .01 and ***p < .001.

As demonstrated in Figure 3(A), treatment with PTX alone (Taxol) or CLPs–PTX–survivin siRNA exhibited a negligible inhibitory effect on the proliferation of U251-CD133^+^ cells, but DP-CLPs–PTX–survivin siRNA treatment demonstrated a prominent inhibition of U251-CD133^+^ cell growth. Furthermore, treatment with Taxol resulted in a high apoptosis percentage in BCECs, but BCEC treatment with either CLPs–PTX–survivin siRNA or DP-CLPs–PTX–survivin siRNA resulted in relatively little apoptosis (Figure 3(B,C)). After treatment with DP-CLPs–PTX–survivin siRNA for 48 h, survivin, nestin, BCRP1 and MGMT protein levels in U251-CD133^+^ cells were significantly decreased. However, GFAP protein levels were significantly increased. Additionally, survivin and nestin protein levels significantly decreased in U251-CD133^–^ cells post-treatment with DP-CLPs–PTX–survivin siRNA for 48 h (Figure 3(D)). It showed that together with the higher internalization of siRNA and PTX, the stronger CD133^+^ glioma cells growth inhibition was observed in DP-CLPs–PTX–siRNA, in agreement with their higher apoptotic ratio. As the combined treatment of survivin siRNA and PTX has been demonstrated to lead to a markedly greater caspase activation and abrogation of the mitotic checkpoint than either survivin siRNA or PTX alone, causing significantly greater levels of cancer cell death. Innate low expression mRNA level of survivin lead to weaker block effect, as the result of reducing the toxicity towards BCECs. Furthermore, by the treatment with DP-CLPs–PTX–survivin siRNA for 48 h, the apoptosis inhibitory protein survivin, neural stem cell marker Nestin and drug resistance-related protein BCRP1, MGMT in U251-CD133^+^ were decreased, but the neural differentiation marker GFAP was increased. It suggested that DP-CLPs–PTX–survivin siRNA facilitated the differentiation of GSCs into non-CSCs, thus ameliorating chemoresistance in U251-CD133^+^ cells.

### *In vivo* antitumor effects

**Figure 4:**
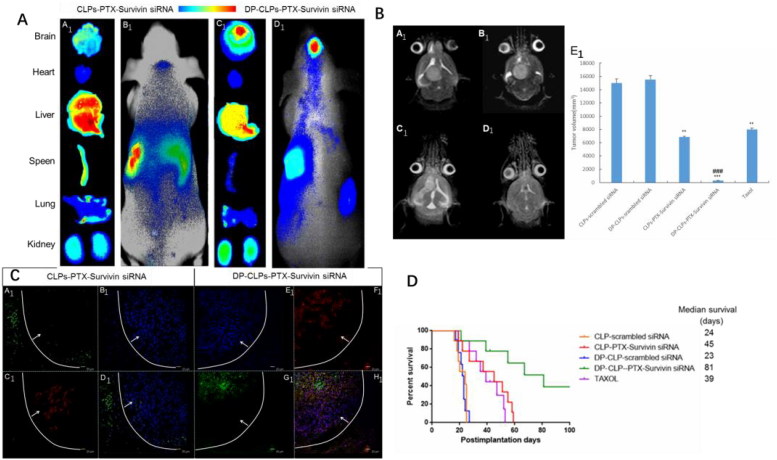
(A) *In vivo* fluorescence imaging of intracranial U251-CD133^+^ glioma tumor-bearing nude mice treated for 24 h with CLPs–PTX–survivin siRNA (B_1_) or DP-CLPs–PTX–survivin siRNA (D_1_) liposomes, as well as corresponding dissected organs (A_1_ and C_1_). (B) Lesions in nude mice implanted in situ with U251-CD133^+^ cells after treatment with CLPs–scrambled siRNA (A_1_), CLPs–PTX–survivin siRNA (C_1_), DP-CLPs–scrambled siRNA (B_1_) or DP-CLPs–PTX–survivin siRNA (D_1_); all lesions were characterized by MRI at 19 days post-injection. U251-CD133^+^ cells were intracranially implanted in nude mice after 48 h treatment with Taxol, CLPs–PTX–survivin siRNA or DP-CLPs–PTX–survivin siRNA. Tumor size was measured 19 days post-injection (*n* = 5/group). **p < .01 versus control, ^###^*p* < .001 versus the CLPs–PTX–survivin siRNA group (E_1_). (C) Evaluation of apoptosis in the brains of intracranial U251-CD133^+^ glioma tumor-bearing nude mice treated with CLPs–PTX–survivin siRNA (A_1_–D_1_) or DP-CLPs–PTX–survivin siRNA (E_1_–F_1_) at 48 h post-administration 2 days after intravenous administration. Images were obtained using an Annexin V-FITC Apoptosis Detection Kit. Image D_1_ is the merge of images A_1_, B_1_ and C_1_; image F_1_ is the merge of E_1_, F_1_ and G_1_. Green: apoptotic cells. Red: CD133. Blue: cell nuclei. White line: glioma border. White arrow: glioma direction. Original magnification: ×100. (D) Survival curve of U251-CD133^+^ glioma tumor-bearing nude mice.

As shown in Figure 4(A), fluorescence signal was much stronger in tumor-bearing brains of DP-CLPs–PTX–survivin siRNA and was much stronger than that of CLPs–PTX–survivin siRNA at 24 h post-injection. Ex vivo evaluation of excised organs and tissues (heart, liver, spleen, lung and kidney) as well as the tumor-bearing brain at 24 h post-injection showed obvious accumulations of DP-CLPs–PTX–survivin siRNA. Although greater CLPs–PTX–survivin siRNA accumulation was detected in the reticuloendothelial system (RES) (which could be interpreted as a result of classical passive targeting), RES uptake could be greatly reduced by the presence of PEG on particle surfaces, as well as by employing the dual ligands modification.

The tumor size at 19 days of the nude mice in situ implanted by U251-CD133^+^+DP-CLPs–PTX–survivin siRNA were much more significantly decreased compared with the group of U251-CD133^+^+Taxol, U251-CD133^+^+CLPs–PTX–survivin siRNA and the control. This finding was consistent with the changes seen on MR images (Figure 4(B)).

Brain tumor apoptosis was assessed at 16 days post-CSC implantation (3 days after the last administration of medicine), CLPs–PTX–survivin siRNA treatment induced tumor apoptosis mainly at the glioma boundary with relatively low efficiency. In contrast, DP-CLPs–PTX–survivin siRNA treatment induced more widespread apoptosis which was present at both the glioma boundary and the tumor interior (Figure 4(C)). Critically, CD133^+^ cell apoptosis was more evident in mice treated with DP-CLPs–PTX–survivin siRNA compared with CLPs–PTX–survivin siRNA-treated counterparts. Presumably, CLPs–PTX–survivin siRNA was able to access gliomal tissues through the EPR effect but could not passively penetrate into the glioma due to increasing tumor tissue density and, thus, could not induce apoptosis inside the glioma. However, DP-CLPs–PTX–survivin siRNA was able to reach the glioma interior and accumulate inside CD133^+^ glioma cells, thus inducing apoptosis in both cells at the edge and the interior of the tumor and especially CD133^+^ glioma cells. This antitumor effect was also reflected in the median survival time of the mice bearing brain tumor xenografts, as those treated with DP-CLPs–PTX–survivin siRNA presented much longer median survival durations relative to CLPs–PTX–survivin siRNA-treated and Taxol-treated groups (Figure 4(D)).

In addition, *in vivo* toxicity of drug delivery system has become a great concern. So we carried out histopathological examination by the staining of organs with H&E. As shown in Supplementary Figure S3, no apparent abnormality or lesion was observed in the heart, lung, liver, spleen and kidney, suggesting that the prepared DP-CLPs may hardly induce treatment toxicity. The results of H&E again confirmed that the as-prepared nanoparticles had little side effects.

## Conclusions

Overall, the prepared DP-CLPs–PTX–siRNA nanocomplex was relatively safe and possessed remarkably targeted therapy of glioma stem cells by combining the chemotherapy of PTX and the small RNA interference effect of survivin siRNA. The promising therapeutic prospect of DP-CLPs–PTX–survivin siRNA may render it a potential approach to improve current cancer treatments, especially for those tumors that have developed a resistance to conventional therapeutic methods.

## Supplementary Material

Supporting_information-drug_delivery.pdf
