# Supplementary material for: Dual-modified cationic liposomes loaded with paclitaxel and survivin siRNA for targeted imaging and therapy of cancer stem cells in brain glioma
Source: Drug Deliv. 2018 Oct 1;25(1):1718–27. doi: 10.1080/10717544.2018.1494225 (PMC6171435; doi:10.1080/10717544.2018.1494225)
Supplement: Supporting_information-drug_delivery.pdf [file IDRD_A_1494225_SM5342.pdf]

Dual-modified cationic liposomes loaded with paclitaxel and survivin siRNA for targeted imaging and therapy of cancer stem cells in brain glioma

Xiyang Sun[a] &Ying Chen[a], Hui Zhao[b], Guanglei Qiao[a], Meiyang Liu[c], Chunlei Zhang[c], Daxiang Cui[c,d], Lijun Ma[a,*]

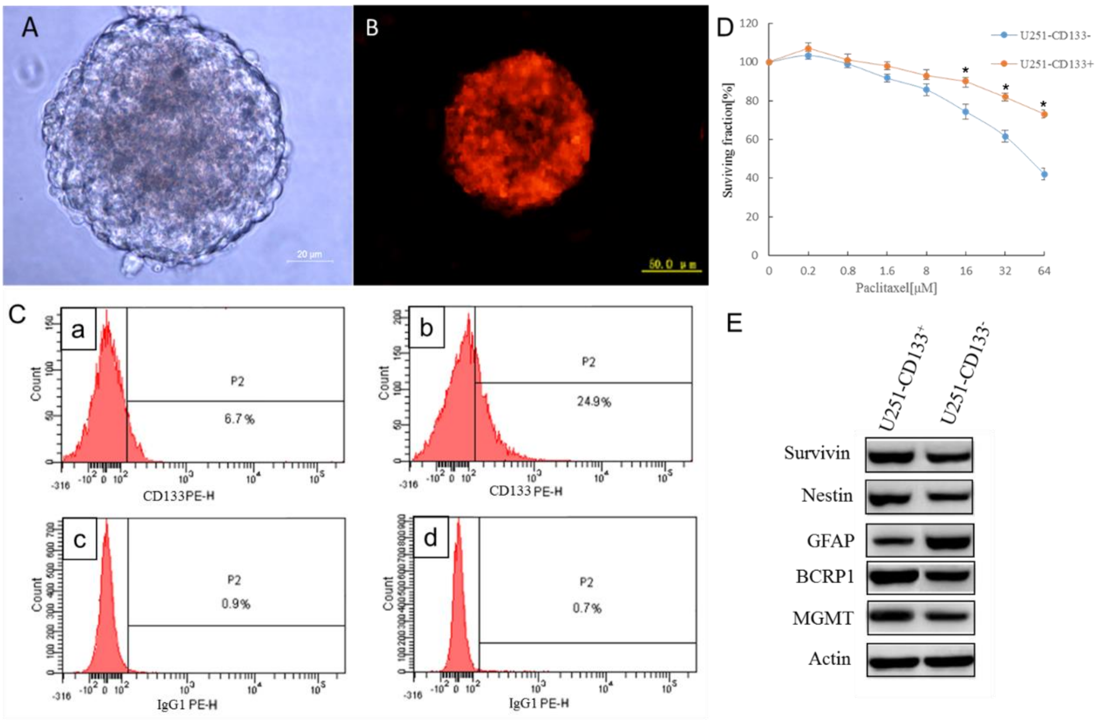

Fig. S1. Isolation and differentiation of U251-CD133$^+$ cells.

A: U251 cells formed tumor spheres after being cultured in STGM for 15 days. B: Tumor sphere immunostaining for CD133 (red). C: The proportion of CD133$^+$ U251 cells after being cultured in (a) DMEM or (b) STGM for 15 days, (c) and (d) were reciprocal isotype controls. D: Drug sensitivity. Both U251-CD133$^+$ and U251-CD133$^-$ cells were collected by FACS sorting and plated in 96-well plates at a density of 1×10^4 cells/well. Cells were then treated with various concentrations of PTX for 24 h. * = $p < 0.05$ compared to autologous CD133$^-$ cells. Data are representative of two independent experiments. E: Survivin, nestin, GFAP, BCRP1, and MGMT protein expression levels as detected by Western blotting.

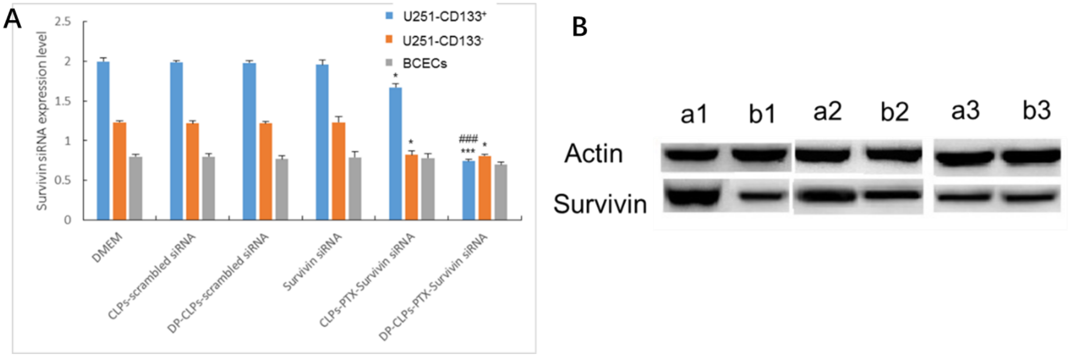

Fig.S2. Survivin mRNA expression.

A: Survivin mRNA expression levels in U251-CD133$^+$ cells, U251-CD133$^-$ cells, and BCECs following treatment with either DMEM, CLPs/scrambled siRNA, DP-CLPs/scrambled siRNA, survivin siRNA, CLPs/PTX/si-survivin, or DP-CLPs/PTX/si-survivin siRNA for 48 h. Indicated values indicate means ± SD (n = 3). The significance of the differences was evaluated using one-way ANOVA followed by Bonferroni test. * = P < 0.05, *** = P < 0.001 versus control, $^{###}$ = P < 0.001 versus CLPs-PTX/si-survivin. B: Western blot analyses on survivin protein expression in U251-CD133$^+$ cells (a1, b1), U251-CD133$^-$ cells (a2, b2), and BCECs (a3, b3) treated with (a) DP-CLPs/scrambled siRNA or (b) DP-CLPs/PTX/si-survivin (b).

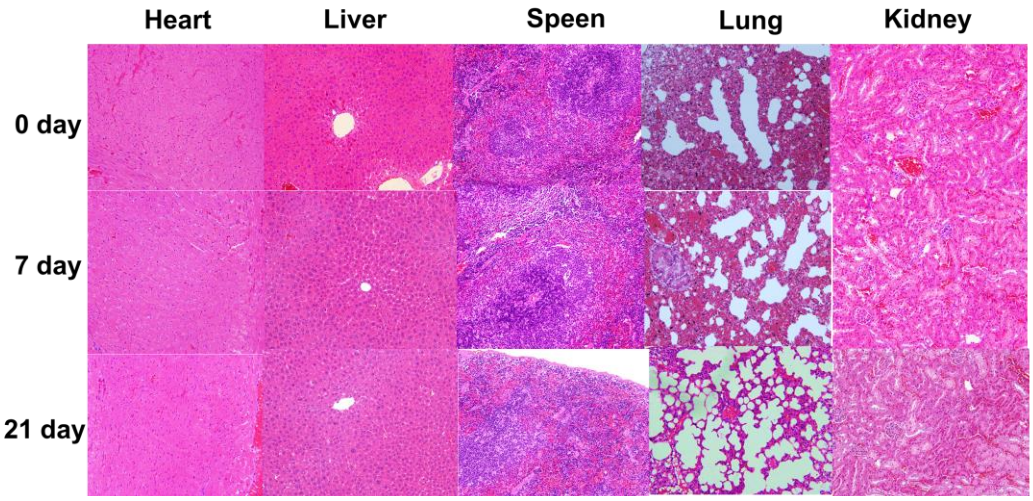

FigureS3. H&E section images of the major organs from the mice after 0, 7, 21 days post-injected with DP-CLPs. Original magnification: x 200.
